# CDK4/6 and PDGFRA Signaling as Therapeutic Targets in Diffuse Intrinsic Pontine Glioma

**DOI:** 10.3389/fonc.2018.00191

**Published:** 2018-05-31

**Authors:** Christine Hoeman, Chen Shen, Oren J. Becher

**Affiliations:** ^1^Department of Pediatrics, Northwestern University, Chicago, IL, United States; ^2^Ann & Robert Lurie Children’s Hospital of Chicago, Division of Hematology-Oncology and Stem Cell Transplant, Chicago, IL, United States

**Keywords:** diffuse intrinsic pontine glioma, K27M, platelet-derived growth factor-B, PDGF-A, CDK4/6, PDGFRA

## Abstract

Diffuse intrinsic pontine gliomas (DIPGs) are incurable childhood brain tumors, whereby the standard of care is focal radiation, a treatment that provides temporary relief for most patients. Surprisingly, decades of clinical trials have failed to identify additional therapies that can prolong survival in this disease. In this conference manuscript, we discuss how genetic engineered mouse modeling techniques with the use of a retroviral gene delivery system can help dissect the complex pathophysiology of this disease. With this approach, autochthonous murine DIPG models can be readily induced to (1) help interrogate the function of novel genetic alterations in tumorigenesis, (2) identify candidate cells of origin for this disease, (3) address how region-specific differences in the central nervous system influence the process of gliomagenesis, and (4) evaluate novel therapeutics in an immunocompetent model.

## Introduction to Diffuse Intrinsic Pontine Glioma (DIPG)

Pediatric brain tumors are now the leading cause of cancer-related death in children (1). Of the various types of pediatric brain tumors, diffuse intrinsic pontine glioma or DIPG stands out as one of the childhood brain tumors with the worse survival with an annual incidence in the United States at 250 cases per year, and a median overall survival of approximately 11 months (2). Children with DIPG usually present at the age of 6–7 years of age, and clinical presentation usually includes the triad of ataxia, cranial nerve palsies, and long tract signs (3, 4). On magnetic resonance imaging or MRI, there is a characteristic T2 hyper intense lesion that is primarily localized in the pons, while T1 with gadolinium imaging demonstrates minimal contrast enhancement. This tumor is highly infiltrative as autopsy examination of the central nervous system of children who succumb to DIPG demonstrates leptomeningeal dissemination in approximately 40% (5). This publication associated with the Alicia Pueyo DIPG workshop that was held in Barcelona on March 12–13, 2018 will review efforts to better understand the pathogenesis of DIPG using genetic mouse modeling techniques. More specifically, we will describe the use of retroviruses to deliver oncogenes or delete tumor suppressors to specific cell populations of the murine neonatal brainstem to induce brainstem gliomagenesis (6). Furthermore, we will describe the application of these brainstem glioma models for functional genomics studies, for investigations of region-specific differences in gliomagenesis within the central nervous system, and for the evaluation of new therapeutics.

## Genetic Alterations

**Figure 1:**
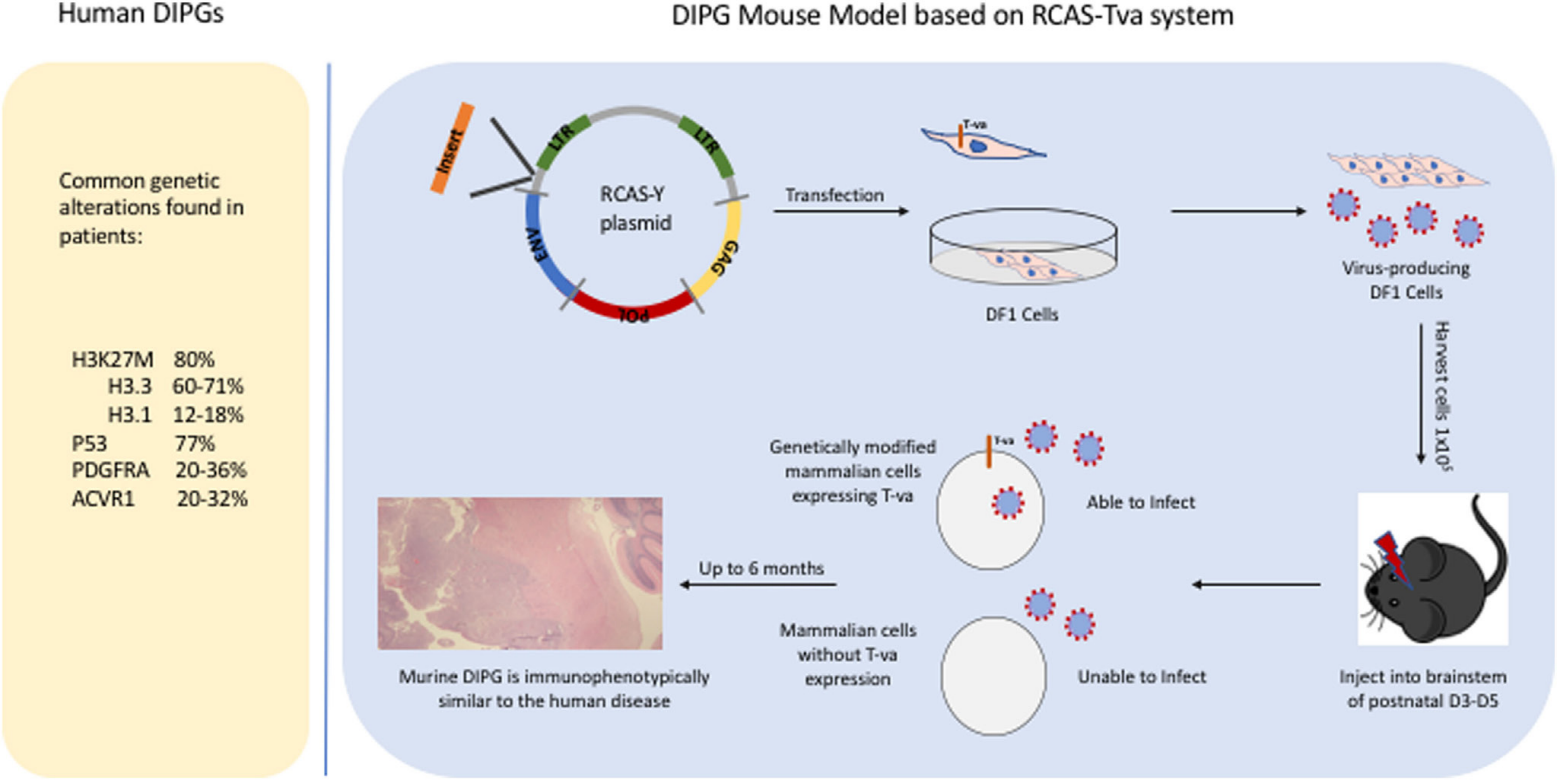
Common genetic alterations in human diffuse intrinsic pontine gliomas (DIPGs) and a schematic illustrating how the RCAS/tumor virus A (TVA) system is used to induce murine DIPGs.

Historically, DIPGs were thought to harbor genetic alterations that are identical to those in adult gliomas, justifying using adult preclinical models to guide the development of novel therapeutics for children with DIPG. With the advent of next-generation sequencing technologies, analysis of DIPGs unraveled new somatic nucleotide variants or SNVs that clearly demonstrate that DIPGs harbor genetic alterations that differ not only from pediatric high-grade gliomas that arise in other parts of the brain, particularly from pediatric high-grade gliomas that arise in the cerebral cortex, but also from adult high-grade gliomas. The SNV discovered in DIPG that has received the most attention so far is the K27M mutation in histone 3 (H3), due to its high prevalence in DIPGs at approximately 80% (and midline gliomas in general) and it being the first time that a cancer-associated SNV was identified in a histone gene itself (7–9). Other SNVs with high prevalence in DIPG (>20%) include activin A receptor, type I (ACVR1), a cell surface receptor in the bone morphogenetic protein pathway, and p53 mutations (10–13). There are also numerous SNVs that have a lower prevalence, such as activating mutations in phosphatidylinositol-4,5-bisphosphate 3-kinase catalytic subunit alpha (PIK3CA) or truncating mutations in protein phosphatase 1D (PPM1D) (14, 15). It is outside the scope of this review to list all SNVs that have been identified in DIPG and readers are referred to a more comprehensive recent analysis published in *Cancer Cell* (16). Besides SNVs, DIPG tumor cells also commonly harbor copy number losses and gains with platelet-derived growth factor receptor A (PDGFRA) being one of the most commonly gained receptor tyrosine kinase and cell cycle regulatory genes harboring focal amplifications in 30% of DIPGs (17, 18). There are also larger genomic alterations, such as whole chromosome arms that are gained or lost in DIPG tumor cells, and the significant of most of these broader genetic changes is less clear as they typically include a large number of genes. Importantly, the H3 K27M mutation, and the ACVR1 mutations are mostly specific to pediatric high-grade gliomas, while mutations in p53, PPM1D, and PIK3CA, as well as PDGFRA gains can also be found in adult high-grade gliomas. Figure 1 lists some of the common genetic alterations that have been identified in human DIPGs.

## Genetic Mouse Modeling

**Figure 2:**
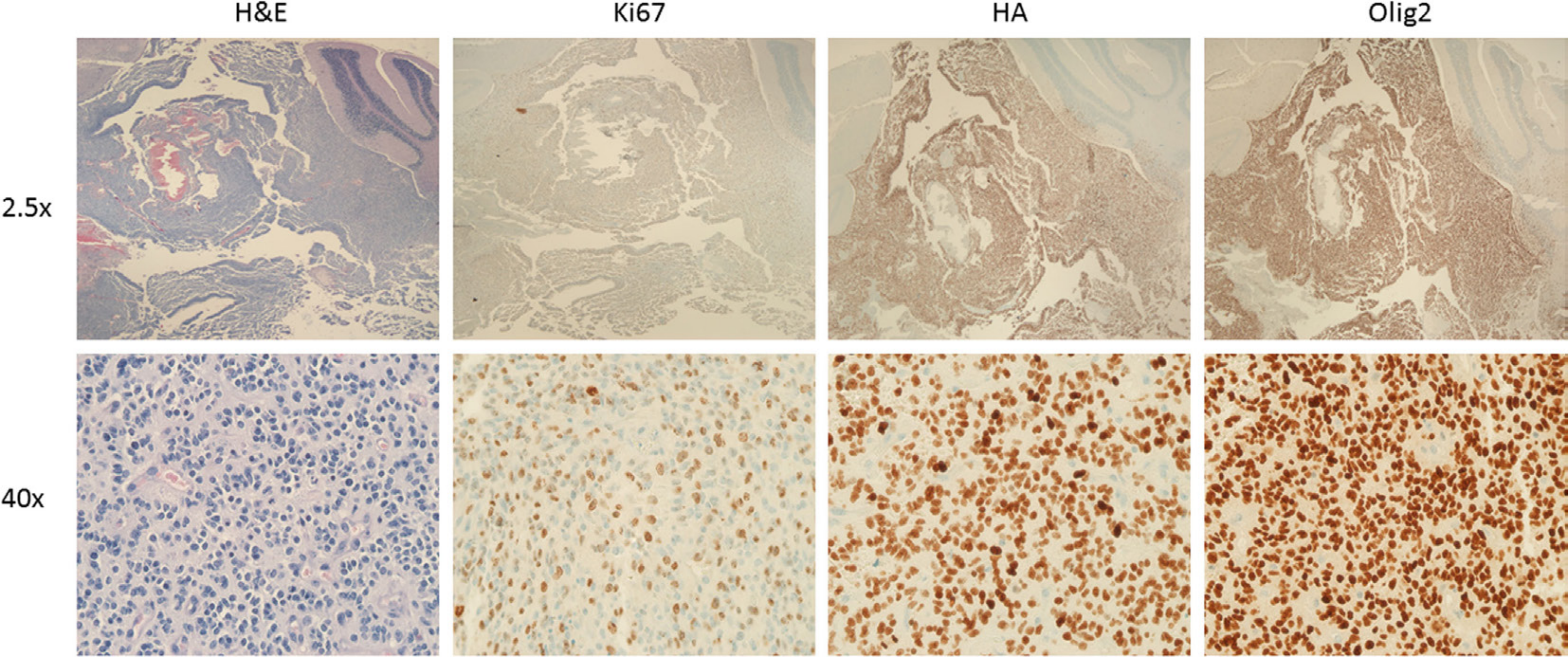
Murine diffuse intrinsic pontine glioma (DIPG) induced by PDGF-A; H3.1K27M; p53 loss. Low (top) and high (bottom) magnification images of a murine DIPG induced with the RCAS/tumor virus A (TVA) system using Nestin TVA; p53 floxed/floxed mice. Neonatal mouse was infected postnatally with DF1 expressing RCAS vectors expressing PDGF-A, H3.1 K27M, and Cre (to delete p53) at a ratio of 1:1:1, and euthanized 43 days’ post-infection. Images from left to right: H&E, Ki-67, HA (tag for H3.1K27M), and Olig2 immunohistochemistry. Note the infiltration into the cerebellum (best seen on low magnification HA and Olig2 immunohistochemistry images).

In this review, we are focusing on the use of the RCAS/tumor virus A (TVA) system to induce murine DIPGs (Figure 1) but it is worth noting that this system has been successful in modeling many other types of cancer including medulloblastomas, sarcomas, and melanomas (19–21). The development of DIPG xenograft models is a great advance that has led to important insights regarding DIPG biology and has been described in detail elsewhere (22, 23). A complementary approach is the development of genetically engineered mouse models which can allow one to investigate questions such as (1) what is the likely cell of origin for DIPG, (2) what is the role of specific oncogene or tumor suppressor in tumorigenesis, and (3) whether a particular genetic event is necessary or sufficient for brainstem gliomagenesis. Before, next-generation sequencing unraveled the presence of H3 mutations in the majority of human DIPGs, there was limited knowledge regarding the genetic alterations in DIPG. We successfully developed a genetic model of DIPG using the RCAS (replication-competent avian sarcoma-leucosis virus long-terminal repeat with splice acceptor)/TVA modeling system by expressing a potent glioma driver, platelet-derived growth factor-B (PDGF-B), specifically in the brainstem (6, 24). By infecting nestin progenitors of the neonatal brainstem with PDGF-B, a ligand that signals through both PDGFRA and PDGFRB, we were able to induce grade II brainstem gliomas that are histopathologically similar to human DIPGs (6). To get higher grade tumors (grades III and IV), we had to combine PDGF-B with either Ink4a-ARF loss or p53 loss (6, 25). In this early study, we demonstrated using immunohistochemistry that PDGFRA is expressed in 67% of human DIPGs. It is worth noting that it is not known whether PDGFB and PDGFRB are expressed in human DIPGs. We have recently observed that we can substitute PDGF-A for PDGF-B with the RCAS/TVA system to induce murine DIPGs (Figure 2) in a similar manner to what has been done to model glioblastomas that arise in adults (26).

Diffuse intrinsic pontine gliomas arises in a unique part of the brain, the pons, a part of the brainstem that harbors critical neurons regulating basic functions such as respiration, heart rate, and blood pressure. This is why surgical resection is not an option for DIPG. One application of genetic mouse models using retroviruses is to interrogate region-specific differences within the central nervous system by generating age- and strain-matched murine gliomas induced by the same genetic events and comparing their differences and similarities using expression profiling. We performed one such experiment with our initial DIPG model driven by PDGF-B and Ink4a-ARF loss. By comparing the expression profile of brainstem gliomas and gliomas induced in the cerebral cortex that were initiated with the same two genetic alterations, we identified a small number of significantly differentially expressed genes. We focused on one such gene, paired box protein 3 or Pax3, a transcription factor that we observed to be upregulated in brainstem gliomas relative to gliomas arising in the cerebral cortex and relative to normal brainstem. Additional experiments demonstrated that pax3 is expressed in tumor cells, that it cooperates with PDGF-B in promoting gliomagenesis, and that a distinct subset of human DIPGs, approximately 40%, also have high expression of pax3 (27).

Accurate genetic mouse modeling of DIPG requires introduction of the genetic alterations to the correct cell of origin during the development of the pons. One challenge is that it is difficult to be certain of the correct cell of origin of the human disease as DIPG is often diagnosed once patients develop sign and symptoms, a relatively late time-point in the natural history of the disease, where the tumor occupies the majority of the pons. With this caveat in mind, there have been a few studies using human postmortem pontine tissue to investigate candidate cell of origin for DIPG. In one study, candidate cells of origin for DIPG were identified as a Nestin+/Vimentin+/Olig2+ cell population located in the ventral pons during middle childhood as this cell population mirrors the age-specific incidence of DIPG (22). In a second study, oligodendrocyte progenitor cells (Olig2+/Sox2+/APC−) were identified as the most proliferative population in the postnatal pons and thus likely cells of origin as proliferating cells are more likely to acquire new mutations during cell division than non-dividing cells, initiating DIPG pathogenesis (28). The RCAS/TVA system allows for investigations into the cell of origin by investigating whether a particular cell of origin can give rise to DIPG when specific genetic alterations are introduced. Thus, far we have induced murine DIPGs in two cells of origin population, namely, nestin-expressing progenitors and pax3-expressing progenitors. While expression of the two markers does overlap in the midbrain and dorsal pons, they have limited overlap in the ventral pons although both are present. In our early work targeting nestin-expressing progenitors, we identified nestin-expressing progenitors lining the floor of the fourth ventricle as putative cells of origin for DIPG (6). However, as the majority of human DIPGs are thought to arise from the ventral pons and it is unclear whether nestin-expressing progenitors lining the floor of the fourth ventricle can give rise to cells in the ventral pons, we also investigated whether pax3 can serve as a cell of origin for DIPG and were successful in generating murine DIPGs, including ones that arise in the ventral pons (29).

One important aspect of DIPG from a therapeutic perspective is its relatively intact blood–brain barrier which is most evident on MRI imaging as DIPGs rarely enhance with gadolinium on T1 sequences. A detailed discussion of the composition of the blood–brain barrier is outside the scope of this communication, but in brief, two main components of the blood–brain barrier are the tight junctions between endothelial cells and efflux pumps such as ABCB1 and ABCG2 that are expressed by endothelial cells and efflux drugs back to the bloodstream. As it is unclear whether it is the tumor location, or its genetic alterations that most contribute to the relatively intact blood–brain barrier of DIPG, we investigated whether the brainstem location or the histone mutation or both that contributes to the relatively intact blood–brain barrier in DIPG. Using the RCAS/TVA genetic mouse modeling approach, we generated murine DIPGs with and without H3.3K27M in both the cerebral cortex and the brainstem, and measured the relative openness of the blood–brain barrier with dynamic contrast-enhanced MRI. Interestingly, we observed that blood–brain barrier permeability was 67% significantly higher in PDGF-B driven gliomas that were induced in the cerebral cortex vs. those that were induced in the brainstem while H3.3K27M did not significantly impact the openness of the blood–brain barrier (30). These results suggest that it is the brainstem location that at least partially contributes to the relatively intact blood–brain barrier of DIPGs but additional studies are required to interrogate whether additional genetic events besides H3.3K27M may contribute to the relatively closed blood–brain barrier in DIPGs.

## Therapeutics (CDK4/6 as a Strategy for Targeting H3K27M)

When the K27M H3 mutations in DIPG were first discovered in 2012, we investigated whether their expression in nestin progenitors of the neonatal brainstem would be sufficient to drive gliomagenesis in a similar manner to PDGF-B. As 20% of human DIPGs do not harbor the H3 mutations but are clinically indistinguishable, it is likely that H3 mutations are not necessary for DIPG formation. Perhaps not surprisingly, expressing the mutant histone alone (H3.3K27M) in nestin progenitors of the neonatal brainstem did not induce tumors by 12 weeks of age. Interestingly, expressing H3.3K27M with p53 loss in nestin progenitors of the neonatal brainstem resulted in the development of ectopic proliferating cell clusters in approximately 70% of the mice by 12 weeks of age but no overt tumors were observed (31). As PDGFRA copy number gains are associated with H3.3K27M, we combined H3.3K27M with PDGF-B, and observed cooperation between the two genetic events. Infection of the nestin-expressing brainstem progenitors with PDGF-B and H3.3K27M resulted in half-of-the-mice developing grade III/IV tumors while similar infections with the combination of PDGF-B with either an empty vector or H3.3 wild-type (WT) resulted in the generation of only grade II tumors (32). In addition, measurement of the proliferation rate of tumors initiated with PDGF-B and H3.3K27M vs. PDGF-B and H3.3 WT or PDGF-B and empty vector demonstrates that H3.3K27M significantly increases the proliferation rate of PDGF-B driven brainstem gliomas. These results suggest that the H3.3K27M requires oncogenic partners to exert its pro-tumorigenic effects in this experimental system.

These results are consistent with experiments performed with neural progenitors derived from human embryonic stem cells where PDGFRA activation was required in combination with H3.3K27M and p53 loss to induce gliomas (33). By contrast, in a more recent study, an *in utero* electroporation using piggyBac transposon-based vectors of H3.3K27M and p53 deletion using CRISPR did succeed in inducing gliomas with these two genetic events suggesting that these two genetic events may be sufficient to drive gliomagenesis in certain settings (34). Surprisingly, the same authors also observed that H3.3K27M expression under the regulation of either the nestin or GFAP promoters in combination with p53 loss was not sufficient to drive gliomagenesis (34).

Going back to the RCAS/TVA system, to address the mechanism by which H3.3K27M increases the proliferation rate of the tumor cells in this model, we performed RNA sequencing (RNAseq) of PDGF-B; H3.3K27M; p53-deficient tumors and PDGF-B; p53-deficient tumors and compared their transcriptome. It is worth noting that we did observe significantly reduced global H3K27me3 in the PDGF-B; H3.3K27M; p53-deficient tumors relative to PDGF-B; p53-deficient tumors in a similar fashion to the reduced global H3K27me3 observed in human DIPGs with K27M H3 mutations (31, 35, 36). We observed approximately 200 differentially expressed genes between the two tumor genotypes. As expected the majority of differentially expressed genes were upregulated in the PDGF-B; H3.3K27M; p53-deficient model relative to the PDGF-B; p53-deficient tumor as the H3.3K27M mutation is thought to contribute to tumor formation through inhibition of the polycomb repressive complex 2 or PRC2, an H3K27 methylase complex that is associated with gene repression. Surprisingly, the RNAseq analysis also identified a short list of transcripts that were repressed by H3.3K27M and some of these were known PRC2 target genes. We chose to focus on one gene in that short list, namely, p16 or ink4a, as it is an endogenous inhibitor of cyclin-dependent kinase 4 and 6 or CDK4/6, two enzymes that regulate the G1 to S cell cycle transition. Through a series of additional experiments, including chromatin immunoprecipitation, we demonstrated that, paradoxically, there is a stronger H3K27me3 signal at the p16 promoter and that pro-tumorigenic mechanism of the H3.3K27M is no longer present when the CDKN2A locus, which codes for both p16 and p19, is deleted. The mechanism for this focal gain in H3K27me3 at the p16 promoter is unclear but has been observed by others including in human DIPG cells (37, 38). Therefore, H3.3K27M represses p16, an endogenous CDK4/6 inhibitor, which suggests that inhibition of CDK4/6 would be a good target to evaluate in H3.3K27M mutant tumor cells, and indeed in *in vitro* studies we observed that PDGF-B; H3.3K27M; p53-deficient cells are more sensitive than PDGF-B; H3.3WT; p53-deficient cells to CDK4/6 inhibition (32).

## Therapeutics (PDGFRA)

As PDGF signaling is a potent driver of brainstem gliomagenesis in myriad DIPG model systems, it is worthwhile to address whether it is a good therapeutic target for DIPG. There have been two early phase clinical trials that enrolled children with DIPG and reported their results regarding the evaluation of small molecule inhibitors targeting PDGFRA and neither inhibitor (imatinib and dasatinib) significantly prolonged survival (39, 40). It would be difficult to determine exactly why neither drugs proved efficacious against DIPG in those two trials as neither studies included a pharmacodynamics assessment in the tumors to determine if the target was inhibited. This is partly due to the delicate location of the tumor in the brainstem making biopsies, a procedure with significant risk to the patient. So unfortunately, these clinical trial failures did not teach us as much as they should. It is definitely possible that the blood–brain barrier played a role in preventing sufficient drug from reaching the tumors with both agents, although there are many other plausible explanations (tumor heterogeneity, feedback loops, etc.). Using our preclinical model, we evaluated dasatinib *in vivo* in the PDGF-B; p53-deficient DIPG model and observed a modest prolongation of survival. Interestingly, the survival benefit with dasatinib was more substantial when we treated PDGF-B; p53-deficient DIPG-bearing mice that were deficient for ABCG2 and ABCB1 (25). Evaluation of dasatinib in human DIPG cell lines *in vitro* have also demonstrated efficacy and as a result there is an ongoing clinical trial in France evaluating the efficacy of dasatinib in DIPG patients (41).

## Conclusion

Genetic mouse modeling of DIPG with the RCAS/TVA system provides a complementary approach to studying human DIPGs to better understand its complex biology with the ultimate goal of developing improved therapies. Increased research efforts focusing on DIPG in the past decade have ushered in new insights regarding DIPG pathogenesis as well as the development of improved DIPG models, both patient-derived and genetic models. Of course, there are many, many questions that remain to be addressed. Is the relatively intact blood–brain barrier the reason for the failure thus far to identify a single drug that can significantly prolong survival in this disease? Is the K27M histone mutation required for tumor maintenance and is it an important therapeutic target? Are some of the epigenetic changes it induces irreversible as has recently been shown for the IDH mutation (42)? Can we harness the immune system to treat DIPG? Would epigenetic drugs show efficacy in children with DIPG as has been suggested in preclinical studies using DIPG xenograft models (23, 43–45)? Ultimately, the acceleration in our understanding of cancer in general and DIPG in particular, together with the availability of new research tools, and increasing neurosurgical expertise in the procurement of tumor biopsies as well as newer approaches such as localized therapy will eventually translate into improved therapies in the not-so-distant future.

## Author Contributions

OB wrote the manuscript. CH and CS prepared figures for the review. All the authors read and approved the submission of the manuscript.

## Conflict of Interest Statement

The authors declare that the research was conducted in the absence of any commercial or financial relationships that could be construed as a potential conflict of interest.

## References

[1] Ostrom QT Gittleman H Liao P Vecchione-Koval T Wolinsky Y Kruchko C CBTRUS statistical report: primary brain and other central nervous system tumors diagnosed in the United States in 2010–2014. Neuro Oncol (2017) 19:v1–88. 10.1093/neuonc/nox158 29117289 PMC5693142

[2] Warren KE. Diffuse intrinsic pontine glioma: poised for progress. Front Oncol (2012) 2:205. 10.3389/fonc.2012.00205 23293772 PMC3531714

[3] Hennika T Becher OJ. Diffuse intrinsic pontine glioma: time for cautious optimism. J Child Neurol (2016) 31:1377–85. 10.1177/0883073815601495 26374787 PMC6025797

[4] Schroeder KM Hoeman CM Becher OJ. Children are not just little adults: recent advances in understanding of diffuse intrinsic pontine glioma biology. Pediatr Res (2014) 75:205–9. 10.1038/pr.2013.194 24192697

[5] Buczkowicz P Bartels U Bouffet E Becher O Hawkins C. Histopathological spectrum of paediatric diffuse intrinsic pontine glioma: diagnostic and therapeutic implications. Acta Neuropathol (2014) 128:573–81. 10.1007/s00401-014-1319-6 25047029 PMC4159563

[6] Becher OJ Hambardzumyan D Walker TR Helmy K Nazarian J Albrecht S Preclinical evaluation of radiation and perifosine in a genetically and histologically accurate model of brainstem glioma. Cancer Res (2010) 70:2548–57. 10.1158/0008-5472.CAN-09-2503 20197468 PMC3831613

[7] Wu G Broniscer A McEachron TA Lu C Paugh BS Becksfort J Jude Children’s Research Hospital-Washington University Pediatric Cancer Genome, somatic histone H3 alterations in pediatric diffuse intrinsic pontine gliomas and non-brainstem glioblastomas. Nat Genet (2012) 44:251–3. 10.1038/ng.1102 22286216 PMC3288377

[8] Schwartzentruber J Korshunov A Liu XY Jones DT Pfaff E Jacob K Driver mutations in histone H3.3 and chromatin remodelling genes in paediatric glioblastoma. Nature (2012) 482:226–31. 10.1038/nature10833 22286061

[9] Khuong-Quang DA Buczkowicz P Rakopoulos P Liu XY Fontebasso AM Bouffet E K27M mutation in histone H3.3 defines clinically and biologically distinct subgroups of pediatric diffuse intrinsic pontine gliomas. Acta Neuropathol (2012) 124:439–47. 10.1007/s00401-012-0998-0 22661320 PMC3422615

[10] Wu G Diaz AK Paugh BS Rankin SL Ju B Li Y. The genomic landscape of diffuse intrinsic pontine glioma and pediatric non-brainstem high-grade glioma. Nat Genet (2014) 46:444–50. 10.1038/ng.2938 24705251 PMC4056452

[11] Buczkowicz P Hoeman C Rakopoulos P Pajovic S Letourneau L Dzamba M Genomic analysis of diffuse intrinsic pontine gliomas identifies three molecular subgroups and recurrent activating ACVR1 mutations. Nat Genet (2014) 46:451–6. 10.1038/ng.2936 24705254 PMC3997489

[12] Fontebasso AM Papillon-Cavanagh S Schwartzentruber J Nikbakht H Gerges N Fiset PO Recurrent somatic mutations in ACVR1 in pediatric midline high-grade astrocytoma. Nat Genet (2014) 46:462–6. 10.1038/ng.2950 24705250 PMC4282994

[13] Taylor KR Mackay A Truffaux N Butterfield Y Morozova O Philippe C Recurrent activating ACVR1 mutations in diffuse intrinsic pontine glioma. Nat Genet (2014) 46:457–61. 10.1038/ng.2925 24705252 PMC4018681

[14] Zhang L Chen LH Wan H Yang R Wang Z Feng J Exome sequencing identifies somatic gain-of-function PPM1D mutations in brainstem gliomas. Nat Genet (2014) 46:726–30. 10.1038/ng.2995 24880341 PMC4073211

[15] Grill J Puget S Andreiuolo F Philippe C MacConaill L Kieran MW. Critical oncogenic mutations in newly diagnosed pediatric diffuse intrinsic pontine glioma. Pediatr Blood Cancer (2012) 58:489–91. 10.1002/pbc.24060 22190243

[16] Mackay A Burford A Carvalho D Izquierdo E Fazal-Salom J Taylor KR Integrated molecular meta-analysis of 1,000 pediatric high-grade and diffuse intrinsic pontine glioma. Cancer Cell (2017) 32:520–37.e5. 10.1016/j.ccell.2017.08.017 28966033 PMC5637314

[17] Zarghooni M Bartels U Lee E Buczkowicz P Morrison A Huang A Whole-genome profiling of pediatric diffuse intrinsic pontine gliomas highlights platelet-derived growth factor receptor alpha and poly (ADP-ribose) polymerase as potential therapeutic targets. J Clin Oncol (2010) 28:1337–44. 10.1200/JCO.2009.25.5463 20142589

[18] Paugh BS Broniscer A Qu C Miller CP Zhang J Tatevossian RG Genome-wide analyses identify recurrent amplifications of receptor tyrosine kinases and cell-cycle regulatory genes in diffuse intrinsic pontine glioma. J Clin Oncol (2011) 29:3999–4006. 10.1200/JCO.2011.35.5677 21931021 PMC3209696

[19] Browd SR Kenney AM Gottfried ON Yoon JW Walterhouse D Pedone CA N-myc can substitute for insulin-like growth factor signaling in a mouse model of sonic hedgehog-induced medulloblastoma. Cancer Res (2006) 66:2666–72. 10.1158/0008-5472.CAN-05-2198 16510586

[20] Kabaroff L Gupta A Menezes S Babichev Y Kandel RC Swallow CJ Development of genetically flexible mouse models of sarcoma using RCAS-TVA mediated gene delivery. PLoS One (2014) 9:e94817. 10.1371/journal.pone.0094817 24733554 PMC3986235

[21] Yang H Kircher DA Kim KH Grossmann AH VanBrocklin MW Holmen SL Activated MEK cooperates with Cdkn2a and Pten loss to promote the development and maintenance of melanoma. Oncogene (2017) 36:3842–51. 10.1038/onc.2016.526 28263969 PMC5501768

[22] Monje M Mitra SS Freret ME Raveh TB Kim J Masek M Hedgehog-responsive candidate cell of origin for diffuse intrinsic pontine glioma. Proc Natl Acad Sci U S A (2011) 108:4453–8. 10.1073/pnas.1101657108 21368213 PMC3060250

[23] Hashizume R Andor N Ihara Y Lerner R Gan H Chen X Pharmacologic inhibition of histone demethylation as a therapy for pediatric brainstem glioma. Nat Med (2014) 20:1394–6. 10.1038/nm.3716 25401693 PMC4257862

[24] Dai C Celestino JC Okada Y Louis DN Fuller GN Holland EC. PDGF autocrine stimulation dedifferentiates cultured astrocytes and induces oligodendrogliomas and oligoastrocytomas from neural progenitors and astrocytes in vivo. Genes Dev (2001) 15:1913–25. 10.1101/gad.903001 11485986 PMC312748

[25] Mittapalli RK Chung AH Parrish KE Crabtree D Halvorson KG Hu G ABCG2 and ABCB1 limit the efficacy of dasatinib in a PDGF-B-driven brainstem glioma model. Mol Cancer Ther (2016) 15:819–29. 10.1158/1535-7163.MCT-15-0093 26883271 PMC4873451

[26] Ozawa T Riester M Cheng YK Huse JT Squatrito M Helmy K Most human non-GCIMP glioblastoma subtypes evolve from a common proneural-like precursor glioma. Cancer Cell (2014) 26:288–300. 10.1016/j.ccr.2014.06.005 25117714 PMC4143139

[27] Misuraca KL Barton KL Chung A Diaz AK Conway SJ Corcoran DL Pax3 expression enhances PDGF-B-induced brainstem gliomagenesis and characterizes a subset of brainstem glioma. Acta Neuropathol Commun (2014) 2:134. 10.1186/s40478-014-0134-6 25330836 PMC4210596

[28] Lindquist RA Guinto CD Rodas-Rodriguez JL Fuentealba LC Tate MC Rowitch DH Identification of proliferative progenitors associated with prominent postnatal growth of the pons. Nat Commun (2016) 7:11628. 10.1038/ncomms11628 27188978 PMC4873968

[29] Misuraca KL Hu G Barton KL Chung A Becher OJ Novel Mouse A Model of diffuse intrinsic pontine glioma initiated in Pax3-expressing cells. Neoplasia (2016) 18:60–70. 10.1016/j.neo.2015.12.002 26806352 PMC4735629

[30] Subashi E Cordero FJ Halvorson KG Qi Y Nouls JC Becher OJ Tumor location, but not H3.3K27M, significantly influences the blood-brain-barrier permeability in a genetic mouse model of pediatric high-grade glioma. J Neurooncol (2016) 126:243–51. 10.1007/s11060-015-1969-9 26511492 PMC4720569

[31] Lewis PW Muller MM Koletsky MS Cordero F Lin S Banaszynski LA Inhibition of PRC2 activity by a gain-of-function H3 mutation found in pediatric glioblastoma. Science (2013) 340:857–61. 10.1126/science.1232245 23539183 PMC3951439

[32] Cordero FJ Huang Z Grenier C He X Hu G McLendon RE Histone H3.3K27M represses p16 to accelerate gliomagenesis in a murine model of DIPG. Mol Cancer Res (2017) 15:1243–54. 10.1158/1541-7786.MCR-16-0389 28522693 PMC5581686

[33] Funato K Major T Lewis PW Allis CD Tabar V. Use of human embryonic stem cells to model pediatric gliomas with H3.3K27M histone mutation. Science (2014) 346:1529–33. 10.1126/science.1253799 25525250 PMC4995593

[34] Pathania M De Jay N Maestro N Harutyunyan AS Nitarska J Pahlavan P H3.3(K27M) cooperates with Trp53 loss and PDGFRA gain in mouse embryonic neural progenitor cells to induce invasive high-grade gliomas. Cancer Cell (2017) 32:684–700.e9. 10.1016/j.ccell.2017.09.014 29107533 PMC5687892

[35] Bender S Tang Y Lindroth AM Hovestadt V Jones DT Kool M Reduced H3K27me3 and DNA hypomethylation are major drivers of gene expression in K27M mutant pediatric high-grade gliomas. Cancer Cell (2013) 24:660–72. 10.1016/j.ccr.2013.10.006 24183680

[36] Venneti S Garimella MT Sullivan LM Martinez D Huse JT Heguy A Evaluation of histone 3 lysine 27 trimethylation (H3K27me3) and enhancer of Zest 2 (EZH2) in pediatric glial and glioneuronal tumors shows decreased H3K27me3 in H3F3A K27M mutant glioblastomas. Brain Pathol (2013) 23:558–64. 10.1111/bpa.12042 23414300 PMC3701028

[37] Chan KM Fang D Gan H Hashizume R Yu C Schroeder M The histone H3.3K27M mutation in pediatric glioma reprograms H3K27 methylation and gene expression. Genes Dev (2013) 27:985–90. 10.1101/gad.217778.113 23603901 PMC3656328

[38] Mohammad F Weissmann S Leblanc B Pandey DP Hojfeldt JW Comet I EZH2 is a potential therapeutic target for H3K27M-mutant pediatric gliomas. Nat Med (2017) 23:483–92. 10.1038/nm.4293 28263309

[39] Pollack IF Jakacki RI Blaney SM Hancock ML Kieran MW Phillips P Phase I trial of imatinib in children with newly diagnosed brainstem and recurrent malignant gliomas: a Pediatric Brain Tumor Consortium report. Neuro Oncol (2007) 9:145–60. 10.1215/15228517-2006-031 17293590 PMC1871662

[40] Broniscer A Baker SD Wetmore C Pai Panandiker AS Huang J Davidoff AM Phase I trial, pharmacokinetics, and pharmacodynamics of vandetanib and dasatinib in children with newly diagnosed diffuse intrinsic pontine glioma. Clin Cancer Res (2013) 19:3050–8. 10.1158/1078-0432.CCR-13-0306 23536435 PMC3685168

[41] Truffaux N Philippe C Paulsson J Andreiuolo F Guerrini-Rousseau L Cornilleau G Preclinical evaluation of dasatinib alone and in combination with cabozantinib for the treatment of diffuse intrinsic pontine glioma. Neuro Oncol (2015) 17:953–64. 10.1093/neuonc/nou330 25534822 PMC5654348

[42] Turcan S Makarov V Taranda J Wang Y Fabius AWM Wu W Mutant-IDH1-dependent chromatin state reprogramming, reversibility, and persistence. Nat Genet (2018) 50:62–72. 10.1038/s41588-017-0001-z 29180699 PMC5769471

[43] Piunti A Hashizume R Morgan MA Bartom ET Horbinski CM Marshall SA Therapeutic targeting of polycomb and BET bromodomain proteins in diffuse intrinsic pontine gliomas. Nat Med (2017) 23:493–500. 10.1038/nm.4296 28263307 PMC5667640

[44] Grasso CS Tang Y Truffaux N Berlow NE Liu L Debily MA Functionally defined therapeutic targets in diffuse intrinsic pontine glioma. Nat Med (2015) 21:555–9. 10.1038/nm.3855 25939062 PMC4862411

[45] Hennika T Hu G Olaciregui NG Barton KL Ehteda A Chitranjan A Pre-clinical study of panobinostat in xenograft and genetically engineered murine diffuse intrinsic pontine glioma models. PLoS One (2017) 12:e0169485. 10.1371/journal.pone.0169485 28052119 PMC5215670

